# Rapidly progressive cognitive impairment resulting in heavy psychosocial burden in a patient with Fabry disease undergoing hemodialysis: a case report

**DOI:** 10.1186/s12882-024-03624-9

**Published:** 2024-06-03

**Authors:** Isao Ohsawa, Akiko Onuki, Fumie Oka, Yoshiyuki Matsuoka, Yuko Makita, Takashi Kobayashi, Yasuhiko Kanaguchi, Yuya Nakamura, Yusuke Suzuki, Yoshikazu Goto, Hiromichi Gotoh

**Affiliations:** 1 Department of Nephrology, Internal Medicine, Saiyu Soka Hospital, 1-7-22, Matsubara, Soka City, Saitama 340-0041 Japan; 2 Department of Rehabilitation, Saiyu Soka Hospital, Soka City, Saitama Japan; 3 Department of Psychiatry, Japanese Red Cross Medical Center, Shibuya-Ku, Tokyo, Japan; 4 Department of Neurosurgery, Saiyu Soka Hospital, Soka City, Saitama Japan; 5 Division of Nephrology, Department of Internal Medicine, Juntendo University Faculty of Medicine, Bunkyo-Ku, Tokyo, Japan

**Keywords:** Fabry disease, Enzyme replacement therapy, Agalsidase alfa, Hemodialysis, Cognitive impairment, Hasegawa dementia rating scale, Mini-mental state examination

## Abstract

**Background:**

Long-term enzyme replacement therapy (ERT) may improve prognosis in the patients with Fabry disease (FD), however, detail psychosocial burden has not been focused on long life expectancy. We experienced a male case of FD under ERT, he was placed on hemodialysis and presented rapidly progressive cognitive function.

**Case presentation:**

A 51-year-old male patient with FD has been receiving ERT from age of 38 years. Hemodialysis was initiated at the age of 47 years. The patient experienced several attacks of cerebral infarction, and brain images demonstrated wide-spread asymptomatic ischemic lesions. His behavior became problematic at the age of 51 years. He often exhibited restlessness during hemodialysis sessions and failure to communicate effectively. The patient experienced impairment of attention and executive function, topographical disorientation, and amnesia. Consequently, it was necessary for medical staff and family members to monitor his behavior for safe extracorporeal circulation and daily life activities. Annual standardized neuropsychiatric testing revealed worsening of cognitive performance.

**Conclusions:**

Despite treating with long-term ERT, it is necessary to determine the psychosocial burden derived from the progression of cognitive impairment in patients with FD undergoing hemodialysis.

## Background

Fabry disease (FD) is a rare X-linked lysosomal storage disease caused by the deficiency or absence of α-galactosidase A (*GLA*) activity. Accumulation of specific glycosphingolipids, such as globotriaosylceramide (Gb3) and digalactosylceramide, in various tissues leads to reduction in the function of multiple organs. Symptoms (e.g., burning pain of extremities, anhidrosis, and diarrhea) are often intolerable, while heart and renal failure might become life-threatening complications. The life expectancy of hemizygous patients with FD is approximately 20 years shorter than that of healthy males [1].

The use of enzyme replacement therapy (ERT) with agalsidase alfa and beta has been the standard therapeutic strategy against FD for > 20 years. Long-term ERT is effective in delaying the progression of concentric left ventricular hypertrophy (LVH) and decline of estimated glomerular filtration rate [2, 3]. Although this specific treatment may improve prognosis, the well-being of a patient remains a challenge. Previous studies using heterogeneous methodological designs revealed that the prevalence of dementia varies considerably (i.e., 0–30%) [4, 5]. Impairment of executive functioning, the speed of information processing, and attention are hallmarks of FD. Involvement of the central nervous system in the late phase of FD leads to cognitive impairment and strongly affects the quality of life of patients [5, 6]. Thus far, the long-term consequences of this condition have not been investigated in detail. Herein, we describe the case of a patient with FD receiving ERT and undergoing hemodialysis, who experienced heavy psychosocial burden due to rapid progressive cognitive impairment.

## Case presentation

**Fig. 1:**
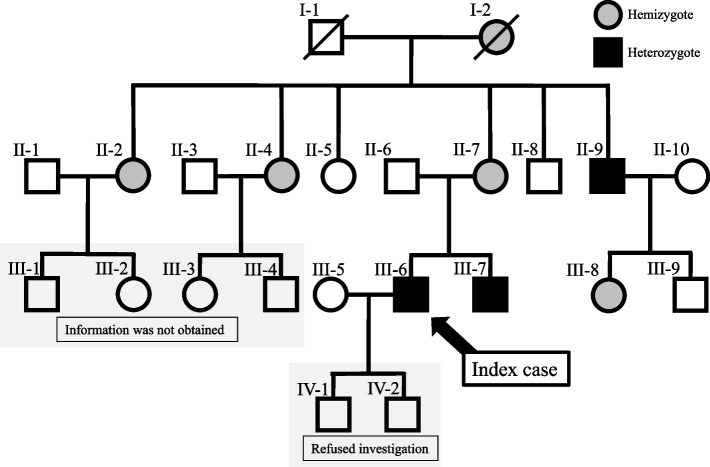
Genealogical tree of the family of this patient. III-6 is an index case of this report. His mother (II-7) is hemizygote and cardiac variant. His uncle (II-9) and his brother (III-7) are classical Fabry disease. His two sons (IV-1 and IV-2) were reluctant to undergo genetic investigation for a definitive diagnosis. Detail information of III-1, III-2, III-3, and III-4was not obtained

A 51-year-old male initially presented with pain in the distal extremities in childhood. At the age of 20 years, a routine annual health check-up revealed LVH and proteinuria. At the age of 37 years, he was definitively diagnosed with classical FD accompanied by renal biopsy findings, including lamellated myelin-like inclusions in the cytoplasm of glomerular and tubular cells, and lower levels of leukocyte *GLA* activity (0.2 nmol/h/mg protein, normal range: 49.8–116.4). At that time, lacunar brain infarction, opacified lens, and angiokeratoma, were also detected. Subsequent investigation revealed that his younger brother was previously diagnosed with classical FD. Moreover, a remarkable family history of genetic disorder was noted (Fig. 1). Specifically, his grandmother died due to stroke and heart disease; three of her four daughter (including the patient’s mother) were heterozygous. His two sons of the patient refused further examination for diagnosis of FD because they had been worried by the peculiar metamorphosed behavior of their father reflecting his psychosocial burden. ERT with agalsidase alfa (Replagal®, 0.2 mg/kg once every 2 weeks) was initiated in this patient from the age of 38 years. A detailed description of the process from diagnosis to the initiation of ERT has been previously published [7].

**Fig. 2:**
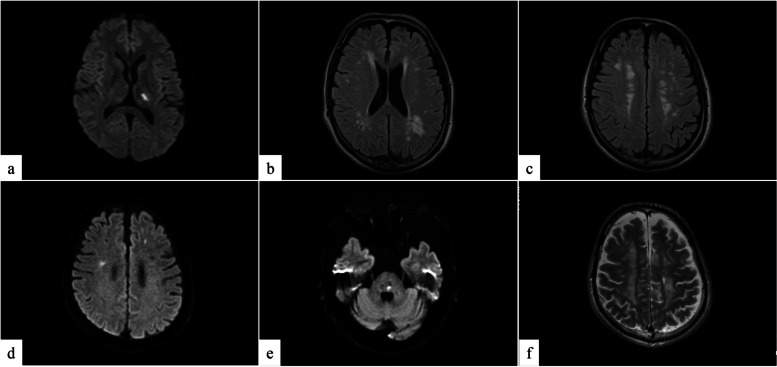
Magnetic resonance imaging. **a** Diffusion-weighted scan and (**b**, **c**) fluid-attenuated inversion recovery (FLAIR): Images revealed a new left internal capsule infarction and multiple asymptomatic brain infarctions at the age of 42 years. **d**, **e** Diffusion-weighted scan and (**f**) T2-weighted scan: the last images of consecutive episodes at the age of 48 years; new infarctions in the bilateral cerebral hemispheres and left pons were detected. There were wide-spread periventricular high-intensity lesions in the parietal cerebral area with different time phases

At the age of 42 years, he experienced a cerebral infarction (left internal capsule) with simultaneous detection of multiple asymptomatic brain infarctions (Figs. 2a–c). Conservative daily treatment with oral medications, namely azilsartan (10 mg), clopidogrel (75 mg), rosuvastatin (2.5 mg), cilostazol (50 mg), and carbamazepine (400 mg), was initiated and did not leave sequalae. The estimated glomerular filtration rate was gradually declined from 65.2 ml/min/1.73 m^2^ at the time of ERT introduction to levels denoting end-stage renal failure. At the age of 47 years, an arteriovenous fistula was created on the left forearm for blood access; however, acute occlusion occurred. After subcutaneously fixing the superficial left brachial artery, hemodialysis was initiated. At the age of 48 years, repeated brain infarction was recorded thrice every 2 months. The first attack was characterized by numbness of the left upper and lower limbs. The second attack was characterized by the numbness of the left upper limb, and magnetic resonance imaging (MRI) revealed right cerebral cortex infarction and micro bleeding. Two months later, MRI demonstrated new infarctions at the left pons and bilateral cerebral cortex without obvious symptoms (Figs. 2d–f). The serial events resulted in permanent damage, which manifested as numbness of the left side of the body and opposite tendon hyperreflexia but no motion impairment. In terms of communication, vocabulary use, and comprehension decreased. In addition, stubbornness and low morale gradually developed, leading to voluntary termination of employment. Thereafter, his daily life was limited to the vicinity of his residence.

At the age of 48 years, the patient continued maintenance hemodialysis at our hospital, which is located near his residence. For the subsequent 3 years, he displayed perplexed utterance and behavior, and his psychosocial burden was raised (Table 1). Due to the impairment of attention and disorientation, it was necessary for medical staff to monitor his behavior before, during, and after each hemodialysis session. He repeatedly interfered with the blood access needle, and, on one occasion, he removed the needle during the hemodialysis session. In addition, disorientation resulted in visiting our hospital at midnight although the next hemodialysis session was scheduled for the next morning. On another day, he was wondering around town after the hemodialysis session and could not return home. He gradually became reclusive, and his family tended to avoid interaction with him. He often expressed objective cognitive complaints, such as awareness of dementia, and appeared conflicted and depressed.

**Table 1:** Psychosocial problems experienced during the past 3 years while undergoing maintenance hemodialysis

Impairment of attention
Interference with the blood access needle, e.g., removal of the needle by himself
Interruption of the hemodialysis session, etc.
Impairment of executive function
Sitting in the locker room for 6 h after hemodialysis session
Walking around in hospital for 3 h after hemodialysis session
Amnesia
Forgetfulness
Inability to adhere to his pharmacological therapy
Aphasia
Decrease in conversation
Failure to communicate effectively
Topographical disorientation
Wondering around, unable to return home
Disinhibition
Poor grooming, changing clothes beside the hemodialysis bed
Agnosia
Not able to find what he is looking for; unable to notice items in front of him
Disorientation
Visiting the hospital on the wrong appointment dates for hemodialysis and clinical examinations
Perplexity, conflict, depression
Social isolation, irritability
Reports of trouble with cognitive progression and financial struggle
Family attitude toward the patient
Tendency to avoid intervention

**Fig. 3:**
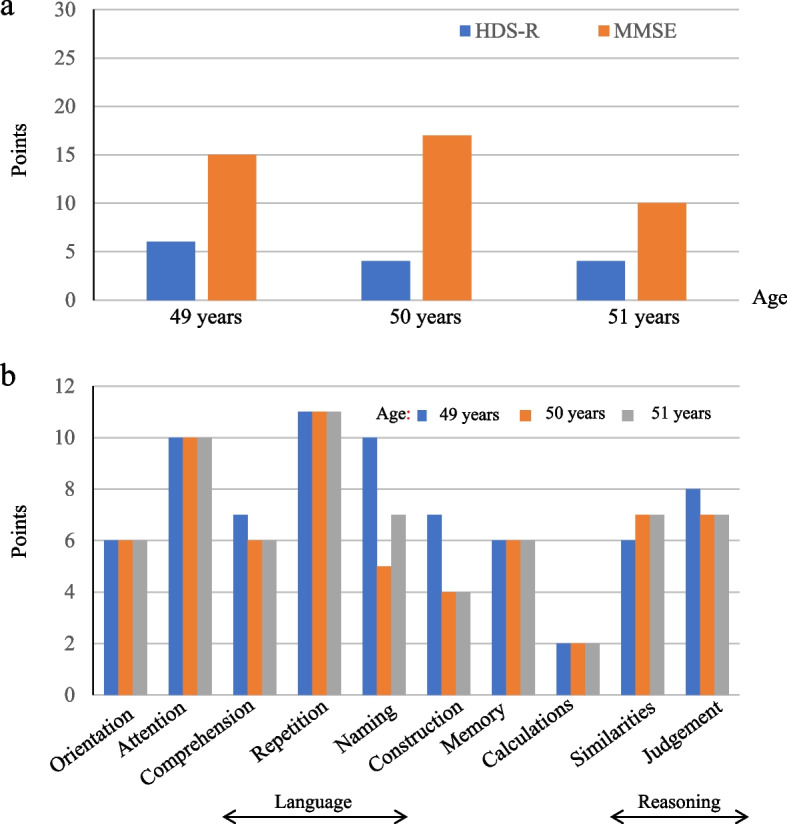
Results of neurophysical examinations of cognitive function. **a** The points of Hasegawa dementia rating scale-revised (HDS-R; maximum: 30 points; blue bar) and mini-mental state examination (MMSE; maximum: 30 points; red bar) demonstrated moderate-to-severe cognitive impairment, which worsened with ageing. **b** Cognitive Status Examination (COGNISTAT) demonstrated severe decline in Orientation, Language (Comprehension, Naming), Construction, Memory, Calculations, and Reasoning (Similarities, Judgement). y.o.: year old

The cognitive function of this patient was monitored on an annual basis using Hasegawa dementia rating scale-revised (HDS-R; maximum: 30 points) and mini-mental state examination (MMSE; maximum: 30 points) at the age of 49, 50, and 51 years (Fig. 3) [8, 9]. The score of HDS-R was 6, 4, and 4 points, while that of MMSE was 15, 17, and 10 points, respectively. Dementia had progressed to severe levels. For a detail analysis of a profile of cognitive performance by domain rather than a global score of functioning, we performed neurobehavioral Cognitive Status Examination (COGNISTAT). This examination was performed on the basis of the instruction provided in the manual [10]. Standardized scores of 10 cognitive domains were obtained, comprising Orientation, Attention, Comprehension, Repetition, Naming, Constructional Ability, Memory, Calculation, Similarities, and Judgement. Scores were standardized as mean = 1 and standard deviation = 1. Lower scores (i.e., 0–9) denoted worse cognitive ability, and scores > 9 were considered normal. Remarkable declines in Orientation, Language (Comprehension, Naming), Construction, Memory, Calculations and Reasoning (Similarities, Judgement) were observed. A relatively good score was maintained for Attention and Language Repetition. Sensitive diagnostic imaging with the Voxel-based Specific Regional Analysis System for Alzheimer’s Disease [11] yielded a Z-score of 0.49, denoting no specific loss of gray matter selectivity in bilateral hippocampal regions. Moreover, the reduction in whole brain gray matter volume was 6.02%, indicating the absence of atrophy despite his multiple brain infarctions.

At the age of 51 years, he received coverage from Public Nursing Care Insurance and began to use short-term residential care at the nursing home for 3 days per week. Gradually, the patient did not show interest in any aspect of life.

## Discussion and conclusions

There is extensive literature on the involvement of multiple organs in FD. However, the clinical problems derived from cognitive impairment in such patients remain elusive [4, 5]. We encountered a case of FD with progressive severe cognitive disorder in a patient aged approximately 50 years undergoing hemodialysis, who experienced a heavy psychosocial burden despite long-term ERT.

Several analyses of brain images of FD revealed that postischemic abnormalities and white matter lesions (e.g., leukoaraiosis) are associated with cognitive impairment [12, 13]. Systemically, Gb3 accumulates in vascular endothelial and smooth muscle cells of patients with FD. In brain tissues, medial thickening, and fibrosis in large vessels, as well as fibrous intimal thickening in medium-sized blood vessels (diameter: 100–1,000 µm) can be observed. In addition, axon swelling, neuronal ballooning, and gliosis are consequences of Gb3 accumulation [14]. Although the present patient did not have obvious brain atrophy (including in the hippocampal regions), these effects typically lead to a general decline in brain function, as a result of the diffuse accumulation of abnormal metabolites in vessel walls and the whole brain.

To our knowledge, there are two reports focusing on patients with FD who developed cognitive impairment during maintenance hemodialysis. Itoh et al. described two male siblings with FD undergoing hemodialysis without ERT [15]. For the younger brother, hemodialysis was introduced at the age of 30 years. At the time of testing for bilateral peripheral vestibular disorder, brain computed tomography revealed ischemic change in multiple periventricular low-density areas. The patient exhibited inconsistent behavior with disorientation and agnosia from the age of 35 years. Brain atrophy and worsening of brain ischemia were also present. Progressive weakness of the extremities resulted in the patient being bedridden. Okeda and Nishihara reported the autopsy of a male case with FD [14]. Peritoneal dialysis was introduced at the age of 36 years. Because of heart failure and peritonitis, he required modal change to hemodialysis at the age of 41 years. Transient confusion was noted, and multiple tiny brain infarctions and wide-spread periventricular high-intensity areas were detected using MRI. At the age of 43 years, hyposomnia and gait ataxia appeared, followed by continuously deterioration of spontaneous speech and memory disturbance. Brain images showed diffuse cerebral atrophy and widespread leukoaraiosis. At the age of 44 years, he was diagnosed with FD, and ERT was initiated 2 years later Nevertheless, the patient expired due to acute myocardial infarction 9 months from the introduction of ERT. Therefore, it is important to regularly check for ischemic changes in the brain and monitor the cognitive function during the long-term observation of FD.

We faced numerous challenges following the introduction of hemodialysis. Often, the patient often could not take an adequate rest during a hemodialysis session. This posed a threat to the safety of extracorporeal circulation; thus, strict observation by medical staff during each hemodialysis session was necessary. Moreover, the patient was unable to visit the hospital and/or return home due to topographic disorientation. Occasionally, medical staff and family members had to search for him. Eventually, his behavior has become unfastidious and calm, he would be bedridden similar to a previously reported case [15]. Cognitive measurement through HDS-R and MMSE revealed a decline in global brain functioning, which progressed with ageing. Using COGNISTAT, we evaluated his profile of cognitive performance by domain rather than by a global score of functioning. Through this approach, we could clearly visualize the deterioration of whole brain performance. Depression is a common psychiatric state in patients with FD, with a prevalence rate ranging from 15% to 62% [5, 13]. Körver et al. conducted a cross-sectional study of 81 patients with FD, demonstrating that the rate of subjective cognitive complaints was markedly higher than that of objective impairments (64% and 16%, respectively) [16]. Their results also indicated a positive correlation between the level of depression, a history of depression, and subjective cognitive complaints. These findings recapitulate that our patient tended to be a hermit and often complained regarding the awareness of his frustration in daily life.

From the perspective of ERT efficacy, Fellgiebel et al. indicated that the size of a white matter lesion mass was positively correlated with LVH [17]. In our case, echocardiography revealed progressive thickening of the posterior wall of the myocardium (i.e., 15, 20, and 26 mm at the age of 40, 50, and 51 years, respectively), with parallel worsening of cognitive impairment. Lyso-Gb3 which is converted from Gb3 by acid ceramidase, is one of blood biomarker of clinical manifestations of FD [18]. Rombach et al. reported that the levels of plasma lyso-Gb3 had positive correlation with the occurrence of white matter lesions in male patients with FD [19]. In this patient, we measured plasma level of lyso-Gb3 by liquid chromatography-tandem mass spectrometry at the age of 52 years [20]. The concentration of lyso-Gb3 (32.7 nmol/L) was markedly higher than the average concentration recorded in normal controls and mass screening of patients undergoing hemodialysis (0.53 nmol/L and 1.7 nmol/L, respectively) [20, 21]. Importantly, long-term ERT was not sufficiently efficacious for pathological suppression. Furthermore, the molecular weight of agalsidase alfa is 102 kDa; thus, this agent is unable to cross the blood–brain barrier. Current knowledge on the efficacy of ERT in the brain is limited.

Research on the family of the patient revealed that three members (II-7, II-9, and III-7) had been treated with ERT. In addition, there are no case of psychological problems or repeated brain infarctions in the II, III and IV generations. Of note, there was no occurrence of young-onset cognitive disorder in this family. We have not addressed the difference in the clinical phenotypes of patients despite harboring the same gene mutation. However, the findings imply that the brain involvement in FD might be affected by multiple factors.

In conclusion, we need to pay attention to the psychosocial burden derived from the progression of cognitive impairment in patients with FD undergoing hemodialysis.

## Data Availability

The datasets used and/or analyzed during the current study are available from the corresponding author on reasonable request.

## Abbreviations

ERT: Enzyme replacement therapy
FD: Fabry disease
Gb3: Globotriaosylceramide
HDS-R: Hasegawa dementia rating scale-revised
LVH: Left ventricular hypertrophy
MMSE: Mini-mental state examination
MRI: Magnetic resonance imaging
